# Shared decision-making in antihypertensive therapy: a cluster randomised controlled trial

**DOI:** 10.1186/1471-2296-14-135

**Published:** 2013-09-11

**Authors:** Iris Tinsel, Anika Buchholz, Werner Vach, Achim Siegel, Thorsten Dürk, Angela Buchholz, Wilhelm Niebling, Karl-Georg Fischer

**Affiliations:** 1 Department of Medicine, Division of General Practice, University Medical Centre Freiburg, Elsässerstr 2m, Freiburg 79110, Germany; 2 Clinical Trials Unit, University Medical Centre Freiburg, Elsässerstr 2, Freiburg 79110, Germany; 3 Institute of Medical Biometry and Medical Informatics, University Medical Centre Freiburg, Stefan-Meier-Str. 26, Freiburg 79104, Germany; 4 Department of Medical Psychology, University Medical Centre Hamburg-Eppendorf, Martinistraße 52, Hamburg 20246, Germany; 5 Department of Medicine, Division of Nephrology, University Medical Centre Freiburg, Hugstetterstr. 55, Freiburg 79106, Germany

**Keywords:** Hypertension, Shared decision-making, Ambulatory blood pressure monitoring, Educational training, Primary care, Family medicine, Cluster randomised controlled trial

## Abstract

**Background:**

Hypertension is one of the key factors causing cardiovascular diseases. A substantial proportion of treated hypertensive patients do not reach recommended target blood pressure values. Shared decision making (SDM) is to enhance the active role of patients. As until now there exists little information on the effects of SDM training in antihypertensive therapy, we tested the effect of an SDM training programme for general practitioners (GPs). Our hypotheses are that this SDM training (1) enhances the participation of patients and (2) leads to an enhanced decrease in blood pressure (BP) values, compared to patients receiving usual care without prior SDM training for GPs.

**Methods:**

The study was conducted as a cluster randomised controlled trial (cRCT) with GP practices in Southwest Germany. Each GP practice included patients with treated but uncontrolled hypertension and/or with relevant comorbidity. After baseline assessment (T0) GP practices were randomly allocated into an intervention and a control arm. GPs of the intervention group took part in the SDM training. GPs of the control group treated their patients as usual. The intervention was blinded to the patients. Primary endpoints on patient level were (1) change of patients’ perceived participation (SDM-Q-9) and (2) change of systolic BP (24h-mean). Secondary endpoints were changes of (1) diastolic BP (24h-mean), (2) patients’ knowledge about hypertension, (3) adherence (MARS-D), and (4) cardiovascular risk score (CVR).

**Results:**

In total 1357 patients from 36 general practices were screened for blood pressure control by ambulatory blood pressure monitoring (ABPM). Thereof 1120 patients remained in the study because of uncontrolled (but treated) hypertension and/or a relevant comorbidity. At T0 the intervention group involved 17 GP practices with 552 patients and the control group 19 GP practices with 568 patients. The effectiveness analysis could not demonstrate a significant or relevant effect of the SDM training on any of the endpoints.

**Conclusion:**

The study hypothesis that the SDM training enhanced patients’ perceived participation and lowered their BP could not be confirmed. Further research is needed to examine the impact of patient participation on the treatment of hypertension in primary care.

**Trial registration:**

German Clinical Trials Register (DRKS): DRKS00000125

## Background

Hypertension is one of the key factors causing cardiovascular diseases (CVD) [1] which make up the most frequent cause of death in industrialised nations [2]. Though effective antihypertensive medication is available, a substantial rate of treated hypertensive patients exceed the (systolic/diastolic) thresholds of 140/90 mmHg in clinical blood pressure measurement (CBPM) and are characterised as uncontrolled (but treated) hypertensive. Rates of controlled treated hypertension (below 140/90 mmHg) range from 43% to 70% in North America [3-8], 49% in Switzerland [9] and between 25% and about 60% in Germany [10-12]. Long term consequences of uncontrolled hypertension like stroke or heart attack lead to high individual and societal burden.

There are two primary reasons to enhance patients’ active participation in medical decisions: (1) Patients have the right to be fully informed on chances and risks of treatments [13,14] and (2) therapies are presumed to be more successful if patients are involved in decisions [15-17]. One concept to achieve this is shared decision making (SDM). SDM interventions are recommended when there are several similarly effective treatment options. This applies to the treatment of uncomplicated arterial hypertension [1,18,19]. Although there is substantial research on SDM, until now there exists little information on the effects of SDM training for general practitioners (GPs) on patient participation *and* clinical outcomes [15-17,20-25]. This holds also for blood pressure (BP) in antihypertensive treatment.

The aim of this study was to implement an evaluated SDM training programme [26] for GPs within the context of hypertension treatment, and to answer the following research questions: Does the SDM training for GPs (1) enhance patients’ perceived participation and (2) lower the BP values of patients?

## Methods

### Trial design and participants

The study was conducted as a cluster randomised controlled trial (cRCT). Each GP practice with its patients was considered as one cluster. As the intervention (SDM training for GPs) affects a GP’s communication style which, in turn, affects all patients of a given GP, a randomisation on patient level would have led to a contamination of treatment conditions. While the intervention took place on GP level, the objectives of the intervention (e.g. enhanced participation and BP decrease) pertained to the patient level. In this study we used ambulatory blood pressure monitoring (ABPM) because of its higher validity compared to CBPM [18,27,28]. Before the first data assessment all participating GP practices were equipped with identical ABPM instruments (Mobil-O-Graph new generation 24h ABP-Control) [29,30]. Under conditions of ABPM a different definition of hypertension is required [1,28,31]. Therefore, in our study any patient is characterised as (uncontrolled) hypertensive whose 24h mean is ≥ 130/80 mmHg or whose daytime mean is ≥ 135/85 mmHg or whose night mean is ≥ 120/70 mmHg.

### Inclusion and exclusion criteria

The eligibility **criteria for GPs** were (1) location in Southwest Germany, (2) offering the full spectrum of a family doctor’s health care services and (3) non-participation in another study implementing an SDM training as intervention. There were no other inclusion or exclusion criteria for GP practices. Most of the participating GPs belonged to teaching practices associated with the Division of General Practice of the University Medical Centre Freiburg (Germany).

Inclusion **criteria for patients** to be screened at T0 were (1) repeated prescription of antihypertensive medication, (2) age of at least 18 years, (3) insured by a statutory health fund with the exception of ‘Bundesknappschaft and See-health insurance’, and (4) understanding of the German language. Exclusion criteria were dementia, mental handicap, or short life expectancy. There were no other inclusion or exclusion criteria for patients.

### Data assessment

Data were collected at four points (T0 – T3). Baseline data (T0) were assessed between June and December 2009. Patients with uncontrolled hypertension (defined as 24h mean ≥ 130/80 mmHg or daytime mean ≥ 135/85 mmHg or night mean ≥ 120/70 mmHg) or with a relevant cardiovascular comorbidity (defined as diabetes mellitus, coronary heart disease [CHD]/heart attack, stroke/transient ischemic attack [TIA] or peripheral arterial occlusive disease [PAOD]) were then included in the study population.

Data of the three follow-up assessments (T1, T2 and T3) were collected at 6, 12 and 18 months (± 2months). The last follow-up measurement (T3) was completed in September 2011. After completing the baseline assessment (T0), GP practices were randomised to either intervention (SDM training) or control group (no training, consultation as usual).

In each of the four data assessments clinical data and self-reported patient data were gathered.

**Clinical data**: Ambulatory blood pressure monitoring (ABPM) was conducted, and values of cholesterol and HbA1c were assessed by the GP practices so that the cardiovascular risk (CVR) of the patients could be calculated.

After each ABPM, the BP protocol was reviewed in a separate consultation and necessary therapy adaptations were decided.

**Self**-**reported patient data**: In each of the four data collections, questionnaires were distributed to the patients by their GP practices. These questionnaires contained a validated German instrument to survey patients’ perceived participation in medical decisions (SDM-Q-9) [32], the translated and validated Medication Adherence Report Scale (MARS-D) [33], and an instrument to assess patients’ knowledge about hypertension (eight items; developed by our research group). Furthermore, the questionnaires referred to changes in antihypertensive therapy and treatment outside the GP practice, to socio-demographic data, tobacco consumption, height and weight. Moreover some other self-reporting instruments [34-37] were included. All scores of the questionnaire instruments with Likert scales were transformed into a scale ranging from 0 (lowest level) to 100 (highest level).

### Intervention

Those GPs who had been allocated to the intervention group took part in an SDM training programme [26] which had been evaluated in various studies [22,23,38,39]. It was adapted to the requirements of antihypertensive treatment in general practice and entailed six hours in total. The training included the following elements: (1) information on arterial hypertension, (2) physician-patient communication and risk communication, (3) the process steps of SDM, (4) motivational interviewing [40,41], (5) introduction of a decision table listing options to lower CVR, and (6) use of case vignettes for role plays simulating physician-patient consultations. Additionally, we recommended implementing a cardiovascular risk calculator for GPs which included elements of SDM [24,42]. Furthermore we delivered patient information flyers [43] to the GPs of the intervention group. The intervention took place before the T1 data assessment.

GPs of the control group treated their patients as usual.

### Outcomes

The outcomes of the study pertained to the patient level. Primary endpoints were (1) change of patients’ perceived participation from T0 to T1, T2 and T3 and (2) change of systolic BP (24h-mean) from T1 to T2 and T3 evaluated as the mean effect over all points in time. Secondary endpoints were (1) change of diastolic BP (24h-mean), (2) change of patients’ adherence, (3) change of cardiovascular risk score (CVR), each from T1 to T2 and T3, and (4) change of patients’ knowledge on hypertension from T0 to T1, T2 and T3. All endpoints were analysed using mixed models [44]. Additionally, the proportion of patients whose BP was uncontrolled at each time point were analysed descriptively.

### Randomisation

To prevent an allocation bias on cluster level and a subsampling bias on patient level, randomisation took place after the baseline assessment [45]. We randomly selected GP practices (including their patients) for the intervention or control group, respectively. The randomisation was conducted by staff members of the Division of General Practice, University Medical Centre Freiburg. No stratification was used. The patients were blinded to the allocation of the intervention (single-blinded study).

### Sample size and power calculation

Power and sample size considerations have been published in the study protocol [46]. In brief, we intended to include 1200 patients in the study population so that after a 20% drop out at the practice level and a 20% drop out at the patient level we could expect to be able to include 788 patients in the final analysis. This would allow detection of effect sizes of 0.3 or 0.35 with a power of 74% or 87%, respectively, assuming an ICC of 0.05 (VIF = 2.15) and using a Bonferroni correction for the two primary outcomes.

### Missing data

The primary endpoints ‘change of perceived participation’ and ‘change of systolic blood pressure’ were analysed according to the intention-to-treat (ITT) principle. According to Kriston et al. [32] missing data in SDM-Q-9 were replaced if there were at most two missing items. Imputation was realised by using the mean of the valid items (on patient level). Subsequently the raw score was calculated and transformed into a scale ranging from 0 to 100. ABPM values were considered valid for a given point in time if ABPM data of at least 7 nocturnal and 14 daytime measurements had been reported. Missing ABPM data were not replaced (for details cf. study protocol [46]). The exploration of the complete dataset showed that for some endpoints, non-response was slightly higher in the control group than in the intervention group. But overall we found no systematic patterns of missing data.

### Statistical analysis of endpoints

Primary and secondary endpoints are defined as change from baseline. For SDM-Q-9 and knowledge on hypertension, the respective T0 data were considered as baseline values, since the intervention may affect both outcomes directly at the first visit after intervention (T1). In contrast systolic and diastolic BP, CVR, and MARS-D cannot be affected by the intervention before the second visit after intervention. Therefore, the respective T1 data were considered as baseline values for these outcomes (for details cf. study protocol [46]). The intervention effect was evaluated as mean effect over all follow-ups using a mixed effects model (adjusted for the respective baseline values of outcomes according to European Medicine Agency (EMA) guideline [47]), where repeated measurements and clustering are dealt with by random effects. All patients contributing a baseline value and at least one follow-up measurement were included in the mixed model analysis.

Two kinds of sensitivity analyses were performed: (1) All endpoints whose baseline values had been calculated from T1 data in the main analysis (BP, CVR, and MARS-D) were re-analysed by using T0 data as baseline. (2) Both primary endpoints were re-analysed adjusting for several prognostic factors (for details on analyses see study protocol [46]). A descriptive analysis was done to illustrate the proportion of patients with uncontrolled (but treated) hypertension at each time point.

Statistical programming was performed with the Statistical Analysis System (SAS) Version 9.2.

### Ethics

Interested GPs and their medical assistants were informed orally and via printed material about the study and data protection. GPs who wanted to take part in the study confirmed via a written consent form. Patients were informed about the study by their GPs. If patients agreed to take part in the study, both patient and GP signed the written informed consent form. The study and the informed consent form were approved by the Ethics Committee of the University Medical Centre Freiburg. The study protocol has been published elsewhere [46].

## Results

### Study procedure

**Figure 1:**
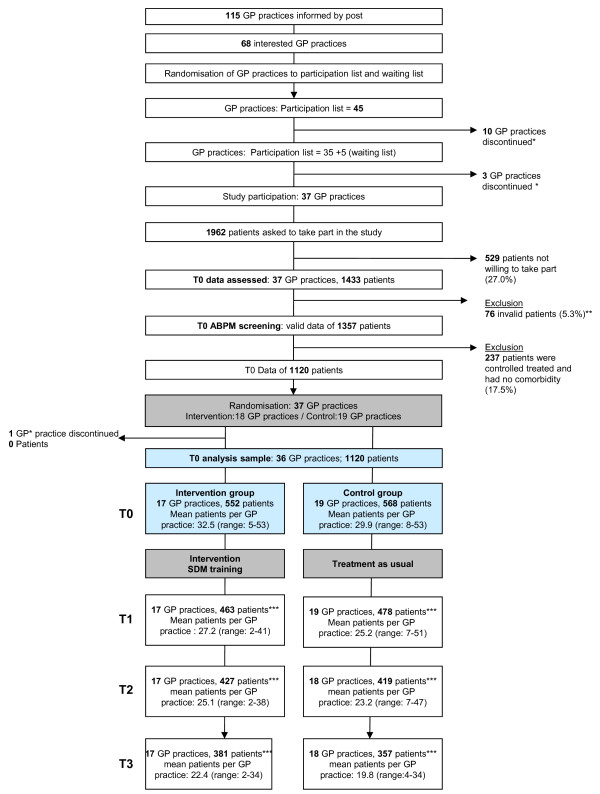
**Recruitment and study procedure including loss to follow**-**up.** GP practices = general practitioner practices. * Details of discontinued GP practices: high work load and lack of staff. ** Details of invalid data: Enrolment in contrary to the inclusion criteria (patients were not member of a statutory health insurance or had not received repeated antihypertensive medication) or questionnaires were missing. *** Details of discontinued patients in both study arms: 1) Intervention group: **171** patients missed at least one follow-up (31.0%). Thereof data of **88** patients are completely lost (15.9%) (loss of T1, T2, and T3) and data of **83** patients (15.0%) are partially available (loss of T1, T2 or T3). 2) Control group: **211** patients missed at least one follow-up (37.1%; including 1 GP practice* with 3 active patients). Thereof data of **86** patients are completely lost (15.1%) (loss of T1, T2, and T3) and data of **125** patients (22.0%) are partially available (loss of T1, T2, or T3). Number of patients available for each analysis is listed in Table 1. Reasons for discontinuation of the patients: patients’ desire (N = 241), change of GP practice (N =18), cardiovascular death (N = 2), other death (N =10), loss of GP practice (3 patients), other cause (N = 65), missing Documentation (N = 41).

The recruitment and study procedure is illustrated in Figure 1. At baseline assessment (T0) we received 1433 BP protocols. The data of 76 patients were excluded because they did not fulfil the inclusion criteria (not a member of a statutory health insurance, no repeated antihypertensive medication) or because of missing questionnaires. Hence data of 1357 patients remained. Then 237 Patients (17.5%) were excluded because they fulfilled two conditions: (1) Their hypertension was characterised as controlled treated (defined as 24h mean < 130/80 mmHg and daytime mean < 135/85 mmHg and night mean < 120/70 mmHg [1,31]) and (2) they had no relevant comorbidity (diabetes mellitus, coronary heart disease [CHD]/heart attack, stroke/transient ischaemic attack [TIA] or peripheral arterial occlusive disease [PAOD]). Hence 1120 patients with uncontrolled but treated hypertension and/or at least one relevant comorbid disorder continued in the study.

Then, the 37 GP practices were randomised to intervention and control group. Immediately before the intervention one GP of the intervention group opted out. This GP practice did not transfer any patient data. In total 17 GP practices received the intervention, 19 GP practices belonged to the control group. These GPs treated their patients as usual. After the T0 assessment was completed and before the T1 assessment started, the intervention (SDM training) took place. In total, the analysis sample included the data of 1120 patients: 552 patients of 17 GP practices in the intervention group and 568 patients of 19 GP practices in the control group. The mean cluster size in T0 amounted to 32.5 patients in the intervention group (range 5–46) and in the control group 29.9 patients (range 8–53); the development of the cluster sizes in the course of the study is shown in Figure 1.

In the intervention group 171 patients missed at least one follow-up measurement. Thereof, 88 patients took part in T0 only and were completely lost to follow up (15.9%). Another 83 patients took part in T0 and in at least one of T1, T2 or T3 (15.0%). Data of the latter patients were included in the mixed models if values for the corresponding endpoints had been available. In the control group 211 patients (37.1%) missed at least one follow-up measurement. Of these, 86 patients were completely lost to follow-up (15.1%). Another 125 patients took part in at least one follow-up measurement so that their data were included in the mixed models (22.0%). The number of patients lost to follow-up is illustrated in Figure 1 and the number of patients available for each analysis is listed in Table 1.

**Table 1:** **Mean changes of endpoints at each follow**-**up to baseline and differences in average mean changes** (**effect estimates**)

**Mean changes**	**Intervention group**			**Control group**			**Difference in average means change from T0/T1 (intervention vs. control)**	**Confidence interval**	**Significance p**
**Number of GP practices**	17	17	17	19	18	18			
**Primary endpoints**									
**SDM**-**Q**-**9**	**T1**-**T0**	**T2**-**T0**	**T3**-**T0**	**T1**-**T0**	**T2**-**T0**	**T3**-**T0**	**T0**		
Valid N	320	287	256	337	272	246			
Mean change	−0.11	−2.875	−1.07	−4.06	−2.34	−3.69	**3**.**1182**	−2.3730; 8.6093	0.2029
(SD)	(21.45)	(22.98)	(21.50)	(22.51)	(20.73)	(20.87)			
**Systolic BP**		**T2**-**T1**	**T3**-**T1**		**T2**-**T1**	**T3**-**T1**	**T1**		
Valid N		414	381		376	337			
Mean change in mmHg		0.59	0.43		−0.02	−0.81	**1**.**7517**	−0.1884; 3.6918	0.0430
(SD)		(11.54)	(12.08)		(10.60)	(10.92)			
**Secondary endpoints**									
**Diastolic BP**		**T2**-**T1**	**T3**-**T1**		**T2**-**T1**	**T3**-**T1**	**T1**		
Valid N		414	381		376	337			
Mean change in mmHg		−0.20	−0.15		−0.15	−0.66	**0**.**9377**	−0.0381; 1.9134	0.0596
(SD)		(6.74)	(6.14)		(6.14)	(5.86)			
**CVR**		**T2**-**T1**	**T3**-**T1**		**T2**-**T1**	**T3**-**T1**	**T1**		
Valid N		294	282		255	212			
Mean change in %		0.71	1.44		1.14	2.04	−**0**.**4891**	−1.4307; 0.4526	0.3084
(SD)		(5.43)	(6.89)		(5.58)	(8.50)			
**MARS**-**D**		**T2**-**T1**	**T3**-**T1**		**T2**-**T1**	**T3**-**T1**	**T1**		
Valid N		360	320		313	287			
Mean change		0.07	0.4		−0.3	−1.1	**0**.**670**	−0.3748; 1.7166	0.2084
(SD)		(9.6)	(10.7)		(6.9)	(6.7)			
**Knowledge**	**T1**-**T0**	**T2**-**T0**	**T3**-**T0**	**T1**-**T0**	**T2**-**T0**	**T3**-**T0**	**T0**		
Valid N	446	402	363	428	369	330			
Mean change	3.00	2.99	5.85	3.30	3.76	4.66	**1**.**3267**	−4.3272; 6.9806	0.6454
(SD)	(27.71)	(28.56)	(28.44)	(30.00)	(28.37)	(24.49)			

*BP* Blood Pressure, *SDM*-*Q*-*9* Shared Decision Making-Questionnaire, *CVR* cardiovascular risk, *MARS*-*D* Medication Adherence Report Scale-German Version.

### Patient characteristics at baseline T0

Patients’ mean age was 63.8 years (± 12.1) in the intervention group, and 65.0 years (± 12.4) in the control group (cf. Table 2). In the intervention group 46.7% of patients were male, compared to 44.7% in the control group. At T0 41.1% of the patients in the intervention group vs. 48.8% of the patients in the control group had at least one relevant comorbidity.

**Table 2:** Patient characteristics at T0

**Characteristics at T0**	**Intervention group**	**Control group**
	**(N =552)**	**(N = 568)**
**Age** mean (SD)	63.8 (± 12.1)	65.0 (± 12.4)
**Gender**: male % (N)	46.7% (258)	44.7% (254)
**Educational level** (Total valid N)	(523)	(521)
low	62.7% (328)	63.8% (332)
middle	24.1% (126)	22.6% (118)
high	13.2% (69)	13.6% (71)
**ABDM** (Total valid N)	(552)	(568)
24h-mean systolic (SD)	133.19 (13.58)	130.80 (12.62)
24h-mean diastolic (SD)	81.98 (9.77)	79.29 (9.74)
**Uncontrolled hypertension**	90.8% (501)	84.7% (481)
≥ **1 Comorbidity**	41.1% (227)	48.8% (277)
Diabetes mellitus	23.7% (131)	32.6% (185)
Heart attack / CHD	16.8% (93)	20.8% (118)
Stroke/ TIA	4.7% (26)	5.5% (31)
Peripheral arterial occlusive disease	3.6% (20)	4.1% (23)
**Manifest arteriosclerosis** (heart attack, CHD, stroke, TIA, or PAOD)	22.6% (125)	26.4% (150)
**CVR score in** % (Total valid N)	(420)	(377)
Mean (SD)	20.40 (± 19.1)	22.85 (± 18.71)
**BMI** (Total valid N)	(545)	(561)
Mean (SD)	28.58 (± 4.78)	28.58 (± 4.79)
**Smoking** (Total valid N)	(544)	(558)
Positive (N)	11.2% (861)	11.6% (65)
**Family history of CVD** (Total valid N)	(528)	(527)
Positive (N)	24.2% (128)	19.4% (102)
**Treatment outside the GP praxis**	35.3% (195)	35.0% (199)
**SDM**-**Q**-9* (Total valid N)	(451)	(489)
Mean (SD)	73.00 (± 17.66)	70.67 (± 20.24)
**Intention**-**to**-**treat**-hypertension scale* (Total valid N)	(521)	(527)
Mean (SD)	80.61 (± 13.32)	79.79 (± 13.52)
**MARS**-D*(Total valid N)	(531)	(539)
Mean (SD)	93.9 (± 9.8)	93.5 (± 10.1)
Knowledge* (Total valid N)	(536)	(547)
Mean (SD)	46.63 (± 27.60)	44.22 (± 28.64)

* Scale 0 – 100 (0 = lowest level and 100 = highest level).

*ABPM* ambulatory blood pressure monitoring, *CHD* coronary heart disease, *TIA* transient ischemic attack, *PAOD* peripheral arterial occlusive disease, *CVR* cardiovascular risk, *BMI* Body Maas Index, *CVD*, cardiovascular disease, *SDM*-*Q*-*9* Shared Decision Making-Questionnaire, *MARS*-*D* Medication Adherence Report Scale-German Version.

Perceived participation (SDM-Q-9) at T0 was relatively high both in the intervention group (mean score 73.00 [± 17.66]) and in the control group (mean score 70.67 [± 20.24]). Patients in both groups reported a very high medication adherence (MARS-D; mean score about 94 in both groups).

The 24h mean systolic / diastolic BP at T0 amounted to 133.2/82.0 mmHg (± 13.6/± 9.8) in the intervention group and 130.8/79.3 mmHg (± 12.6/± 9.7) in the control group. Almost 90.8% of the patients (N = 501) in the intervention group and 84.7% of patients (N = 481) in the control group exceeded at least one of the six ABPM thresholds (24 h or day or night), i.e. these patients were characterised as ‘uncontrolled treated’. The 24h mean systolic BP of patients with uncontrolled hypertension in the intervention group amounted to 134.9 mmHg (± 12.8) and for the control group to 133.5 mmHg (± 11.5). The 24h mean systolic BP of all patients characterised as controlled hypertensive but with relevant comorbidity (12.2%, N = 138) was considerably lower and amounted to 116.0 mmHg (± 7.2) in the intervention group and to 115.8 mmHg (± 6.7) in the control group.

After the first ABPM and the following adaptation of the therapy in T0, the mean BP values (systolic/diastolic) in the whole sample decreased by 3.4/2.4 mmHg to 127.9/78.0 mmHg (±12.2/±9.4) at T1. This decrease cannot result from the intervention because the intervention could have affected BP values after the T1 consultation at the earliest. The baseline BP value (T1) in the intervention group amounted to 128.9/79.2 mmHg (±12.5/±9.5) and in the control group to 127.0/76.8 mmHg [±11.8/±9.1) (cf. Table 3). Even though the mean BP values in both study arms were below recommended BP values for ABPM in T1, the proportion of patients with uncontrolled (but treated) hypertension (as defined: at least one of the six ABPM thresholds was exceeded) in the intervention group amounted to 76.9% (N = 356) and in the control group to 71.6% (N = 327) (cf. Table 4).

**Table 3:** Patient characteristics at each assessment point

	**Intervention group**				**Control group**			
	**T0**	**T1**	**T2**	**T3**	**T0**	**T1**	**T2**	**T3**
**Number GP practices**	17	17	17	17	19	19	18	18
**Primary endpoints**								
**SDM**-**Q**-**9**								
Total valid Nr	451	363	333	301	489	368	295	269
Mean	73.00	73.03	70.51	71.71	70.67	66.55	67.20	66.60
(SD)	(17.66)	(19.54)	(20.98)	(20.59)	(20.24)	(21.34)	(20.00)	(20.71)
**Systolic BP**								
Total valid N	552	463	418	383	568	457	394	348
Mean in mmHg	133.19	128.86	129.27	128.68	130.80	126.98	126.62	125.98
(SD)	(13.58)	(12.51)	(12.80)	(11.42)	(12.62)	(11.75)	(12.29)	(11.23)
**Secondary endpoints**								
**Diastolic BP**								
Total valid N	552	463	418	383	568	457	394	348
Mean in mmHg	81.98	79.21	79.07	78.55	79.29	76.75	76.75	75.64
(SD)	(9.77)	(9.49)	(8.79)	(8.81)	(9.74)	(9.10)	(9.10)	(8.46)
**CVR**								
Total valid N	420	364	320	312	377	336	270	233
Mean in %	20.40	21.02	22.33	23.92	22.85	23.54	24.03	24.34
(SD)	(18.15)	(18.61)	(18.82)	(19.10)	(18.71)	(19.19)	(19.23)	(19.60)
**MARS**-**D**								
Total valid N	531	419	387	349	539	408	357	326
Mean	93.9	95.4	96.0	96.1	93.5)	96.0	96.1	95.3
(SD)	(9.8)	(8.8)	(6.8)	(8.0)	(10.1)	(6.3)	(6.8)	(7.5)
**Knowledge**	536	455	412	371	547	444	382	343
Total valid N	536	455	412	371	547	444	382	343
Mean	46.63	50.05	50.18	53.34	44.22	47.04	47.64	48.51
(SD)	(27.60)	(28.39)	(28.32)	(28.21)	(28.64)	(30.11)	(30.49)	(30.15)

*BP* Blood Pressure, *SDM*-*Q*-*9* Shared Decision Making-Questionnaire, *CVR* cardiovascular risk, *MARS*-*D* Medication Adherence Report Scale-German Version.

**Table 4:** **Patients with uncontrolled** (**but treated**) **blood pressure from T0 to T3**

Uncontrolled BP*: percentage	**Intervention group**	**Control group**
**T0** Total valid numbers N	552	568
**Uncontrolled BP** Valid percentages % (number N )	90.8% (501)	84.7% (481)
**T1** Total valid numbers N	463	457
**Uncontrolled BP** Valid percentages % (number N )	76.9% (356)	71.6% (327)
**T2** Total valid numbers N	418	394
**Uncontrolled BP** Valid percentages % (number N )	78.2% (327)	69.0% (272)
**T3** Total valid numbers N	383	348
**Uncontrolled BP** Valid percentages % (number N )	78.3% (300)	69.8% (243)

*Uncontrolled BP: if 24h-mean ≥ 130/80 mmHg or day-mean ≥ 135 /85 mmHg or night-mean ≥ 120/70 mmHg.

### Effects of the SDM training on primary endpoints

#### Effect on patients’ perceived participation (SDM-Q-9)

**Figure 2:**
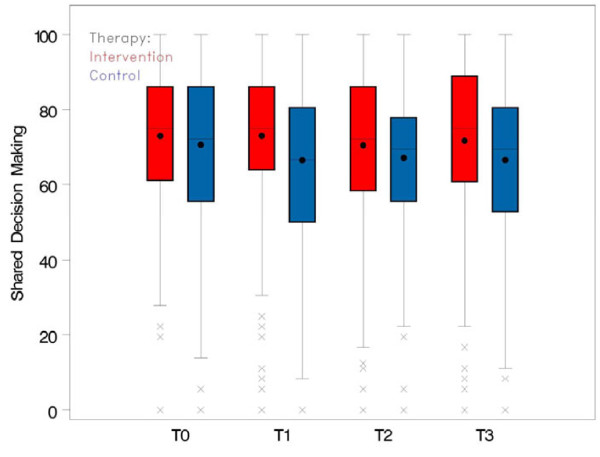
**Perceived participation measured by SDM-Q-9 in intervention and control group from T0 (baseline) to T3.**

**Figure 3:**
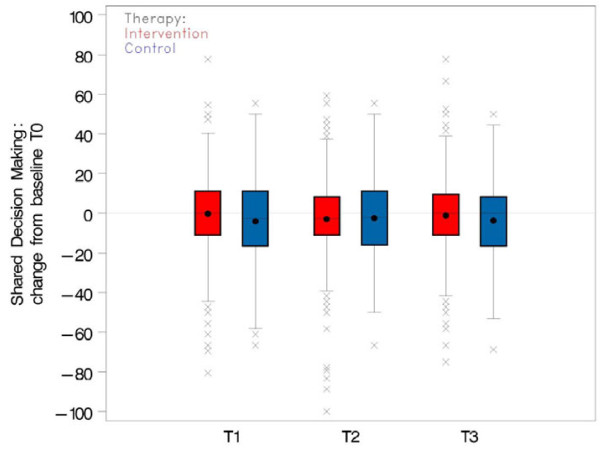
**Average change of SDM-Q-9 from T1, T2, and T3 relative to T0 (baseline) in intervention and control group.**

At baseline assessment (T0), the intervention group had a slightly higher mean SDM-Q-9 score than the control group (cf. Figure 2). The mean SDM-Q-9 score in the control group decreased after baseline assessment. The patients of the control group perceived the highest participation after the first consultation reviewing the ABPM protocol. In the intervention group the mean SDM-Q-9 score remained approximately on the same level at T1 but it also decreased slightly after T1 (cf. Figure 3). After the intervention, the average decrease of the SDM-Q-9 score was slightly smaller in the intervention group than in the control group. According to the mixed model analysis, the average change from T0 was 3.11 points higher in the intervention group than in the control group (97.5% CI [−2.37; 8.61], p = 0.203). The effect was not significant at the (Bonferroni-corrected) 2.5% level (cf. Table 1).

#### Effect on systolic blood pressure

**Figure 4:**
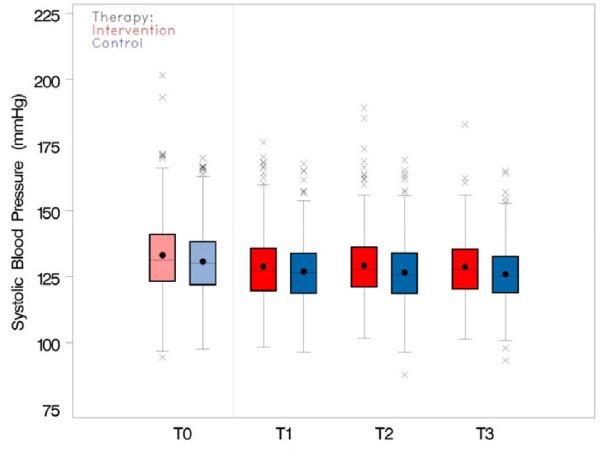
**Systolic blood pressure from T0 (screening value), and T1 (baseline) to T3 in intervention and control group.**

**Figure 5:**
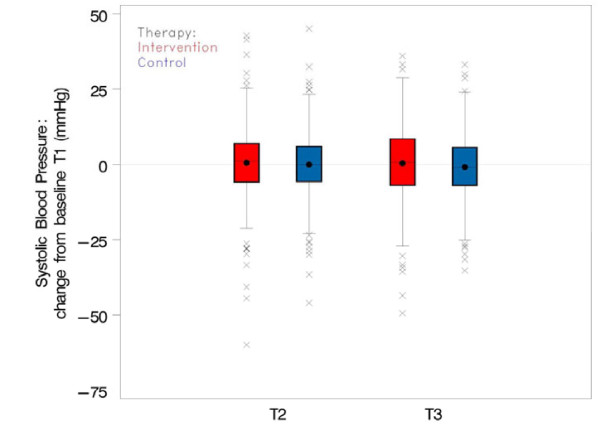
**Average change of systolic blood pressure from T2 and T3 relative to baseline (T1) in intervention and control group.**

In all four data assessments the mean systolic BP was slightly higher in the intervention group than in the control group (cf. Figure 4 and Table 3). Distinct decreases in BP could be observed in both study arms between T0 and T1, i.e. after the initial assessment, and a possible change in patient’s treatment (cf. Figure 4). BP measurements at T2 and T3, which were expected to be affected by the SDM training, did not show relevant changes compared to T1 (cf. Figure 5). The average change in BP from T1 was – according to the mixed model analysis – higher in the intervention group than in the control group (effect estimate +1.75 mmHg (97.5% CI [−0.189; 3.69], p = 0.043). This resulted from a slight BP decrease in the control group and a slight BP increase in the intervention group (cf. Table 1). The comparative increase of systolic BP in the intervention group was against our expectations. However, it is not significant at the level of 2.5%. The sensitivity analyses with (1) baseline T0 and (2) 12 additional prognostic factors showed similar trends with average intervention effects of +1.07 mmHg (97.5% CI [0.76; 2.90]) and +2.39 mmHg (97.5% CI [0.23; 4.53]), respectively.

In summary, the analyses showed unexpected trends: (1) the positive (but not significant) intervention effect on participation resulted from a decreased participation in the control group rather than an increase in the intervention group. And (2) a comparative (but not significant) increase of systolic blood pressure was observed in the intervention group compared to the control group.

#### Effects of the SDM training on secondary endpoints

The mean changes in secondary endpoint variables were very small, and there was no significant intervention effect on any of these endpoints (at significance level 5%). The intervention led to a negligible decrease of 0.49% in CVR (95% CI [−1.43; 0.45], p = 0.308) in the intervention group compared to the control group. The knowledge of the patients about hypertension increased slightly in both study arms. However, the mean intervention effect amounted to only +1.33 points (95% CI [−4.33; 6.98], p = 0.645) and was not significant. The mean adherence score (MARS-D) was very high at the beginning of the study (cf. Table 3), thus leaving little potential for improvement. The mean intervention effect (cf. Table 1) amounted to only 0.67 points (95% CI [-0.37; 1.72], p = 0.208).

## Discussion

### Main results

The results of this study showed that the SDM training programme for GPs neither enhanced patients’ participation significantly nor contributed to a BP decrease, to a higher adherence or knowledge. The CVR-lowering effect of the SDM training amounted to 0.49% (p = 0.308), was negligible and not significant. Deviating from our hypothesis the SDM training did not show any relevant effect on primary or secondary endpoints. Instead, the implementation of the ABPM initiated an improvement of the hypertension treatment, followed by a mean BP decrease between T0 and T1 by (systolic/diastolic) 3.4/4.4 mmHg (±12.2/±9.4) in all patients.

### Strengths and weaknesses of the study

Because most of the participating GPs were academic family doctors associated with the Division of General Practice of the University Medical Centre Freiburg (Germany), the external validity of our results seems to be limited. Therefore we may assume that the participating GPs are probably more open-minded as to taking part in studies and improving their skills when compared to all GPs in the region. Thus, e.g., we assume that GPs who were interested in improving their antihypertensive treatment or their communication skills were overrepresented in this study.

The internal validity of the study is limited to some extent because the intervention itself could not be blinded for the GPs. With respect to the endpoint ‘patients’ perceived participation’ our study might also be internally limited: Recent discussions about the SDM-Q-9 instrument have raised doubts about its content validity [48,49]. Nevertheless the internal validity of the data seems to be fairly high. The loss to follow-up in primary care studies with long term follow up is higher than in clinical studies and was taken into consideration in our sample size calculation. In both arms of this study about 15% of the patients were completely lost to follow-up (15.9% in the intervention group and 15.1% in the control group). Another 15% in the intervention group and 22% in the control group were lost at one or two of the three follow-ups. In the power calculation we estimated that 788 patients would finish the T3 assessment – and this number was nearly achieved (N = 738). In total the missing patterns were rather similar in both study arms. Additionally, the implementation of a cluster-randomised design and a rigorous statistical analysis reinforce the evidence of the results. Therefore the results of our study seem sufficiently valid.

It would have been desirable to evaluate to what extent the GPs in the intervention group really implemented a communication style in accordance with the SDM principles. As we measured only patients’ perceptions on the subject, we do not know to what extent the GPs of the intervention group changed their behaviour ‘objectively’.

### Comparison with other studies and interpretation

In comparison with other studies in which an SDM intervention had been effective [15,17,24] our GP training was rather short. Reviews investigating the effects of SDM interventions ascertain that it is often difficult to bring about behavioural change in doctors with short training programmes [20,21]. Thus one could argue that a more intensive training or other dissemination strategies like coaching on the job or internet-driven feedback strategies might be useful to change GPs’ communication behaviour. However in the study by Deinzer et al. [25], which was characterised by an SDM programme for GPs with a 16h communication training plus supervision, the intervention neither enhanced participation nor improved BP control. A Cochrane review from 2010 analysed various educational training programmes (not confined to SDM training) directed to physicians to improve antihypertensive treatment and did not find any effective training which significantly lowered patients’ BP [50]. A recent review concluded that to date there were no consistent results regarding the effectiveness of patient participation on health-related outcomes [51].

From prior process evaluations of our SDM training we may presume that the intervention was sufficiently accepted by the participating GPs. An intensive and ‘invasive’ training or a direct evaluation of consultations, however, would probably have affected GPs’ acceptance of the intervention and the feasibility of the study.

Our finding that BP decreased between T0 and T1 is in line with other studies which showed that the implementation of ABPM contributed to a more effective antihypertensive treatment [52-54]. However, it is necessary to take into consideration that at T0 the mean 24h BP values of the whole sample only marginally exceeded the recommended ABPM thresholds. Moreover, the consultation that immediately followed the ABPM at T0 provided the opportunity to GPs and patients to optimise their antihypertensive therapy with all usual treatment options in both study arms before the SDM training for GPs started. Accordingly, in T1 the mean 24h BP values were even below the ABPM diagnostic thresholds in both study arms. This might indicate that systolic or diastolic BP values of most patients whose hypertension was characterised as treated but uncontrolled in T1 (intervention group: 76.9% [N = 356], control group: 71.6% [N = 327], cf. Table 4) probably exceeded the recommended ABPM thresholds – e.g. at night – only marginally. Taking into account these circumstances, aggressive treatment escalations could not be expected for the majority of the study population after T1. Therefore one could expect only relatively minor treatment responses to further treatment changes on mean BP.

## Conclusion

Considering that a distinct BP decrease could be noticed in both study groups at the very beginning of the study, i.e. between T0 and T1, we may put forward the hypothesis that the implementation of ABPM can lead to a therapeutic adaption with a positive effect on BP optimisation. The subsequent implementation of a rather short SDM training for GPs does obviously not increase the effectiveness of such a therapeutic adaptation regarding BP decrease.

Though well accepted by the GPs, our results seem to discourage the optimistic expectation that SDM training for GPs solely leads to a relevant increase in patients’ perceived participation or an improvement of clinical outcomes. As patient participation is established in law in Germany, further research should be undertaken to examine the process of patient participation in primary care.

## Abbreviations

ABPM: Ambulatory blood pressure monitoring/measurement; BP: Blood pressure; CBPM: Clinical blood pressure measurement; CHD: Coronary heart disease; cRCT: Cluster randomised controlled trial; CVD: Cardiovascular disease; CVR: Cardiovascular risk; DFG: German Research Foundation; EMA: European medicine agency; GP: General practitioners; ICC: Intra-cluster correlation; ITT: Intention-to-treat; MARS-D: Medication adherence report scale - German version; PAOD: Peripheral arterial occlusive disease; SAS: Statistical analysis system; SDM: Shared decision making; SDM-Q-9: 9-item-questionnaire measuring patients’ perceived participation in medical decision making; TIA: Transient ischaemic attack.

## Competing interests

The authors declare that they have no competing interests.

## Authors’ contributions

KGF, WN, TD, IT conceived the study. IT was responsible for the realisation of the project and its data management. AB and WV carried out the mixed model analyses. AS, TD, IT, AB, and WV interpreted the results. All authors were involved in drafting the manuscript and revised it critically. All authors read and approved the final manuscript.

## Authors’ information

IT^1^: Sociologist (M.A.), coordinator of the study.

AB^2^: Statistician (diploma and PhD). Biostatistician at the Clinical Trials Unit and the Institute of Medical Biometry and Medical Informatics at the University Medical Centre Freiburg.

WV^3^: Statistician (diploma and PhD) and Professor of medical informatics and clinical epidemiology at the University of Freiburg.

AS^1^: Social scientist (PhD and MPH).

TD^1^: Assistant physician, MD (Dr. med.) and researcher.

WN^1^: General practitioner, MD (Dr. med). Professor and head of the Division of General Practice at the University Medical Centre Freiburg, Germany. Board member of the commission of the German Agency for Quality in Medicine (AQuMed/AEZQ), decision board member of the German Medical Association and co-editor of the German Journal of Family Medicine (ZFA).

AB^4^: Psychologist (diploma and PhD), senior researcher.

KGF^5^: Nephrologist, MD (Dr. med). Working with the Division of Nephrology, University Medical Centre Freiburg, and a dialysis centre in Lörrach, Germany.

## Pre-publication history

The pre-publication history for this paper can be accessed here:

http://www.biomedcentral.com/1471-2296/14/135/prepub
